# The I2020T Leucine-rich repeat kinase 2 transgenic mouse exhibits impaired locomotive ability accompanied by dopaminergic neuron abnormalities

**DOI:** 10.1186/1750-1326-7-15

**Published:** 2012-04-25

**Authors:** Tatsunori Maekawa, Sayuri Mori, Yui Sasaki, Takashi Miyajima, Sadahiro Azuma, Etsuro Ohta, Fumiya Obata

**Affiliations:** 1 Division of Clinical Immunology, Graduate School of Medical Sciences, Kitasato University, 1-15-1 Kitasato, Minami-ku, Sagamihara, Kanagawa 252-0373, Japan; 2 Division of Clinical Immunology, Graduate School of Medical Sciences, Kitasato University, 1-15-1 Kitasato, Minami-ku, Sagamihara, Kanagawa 252-0373, Japan; 3 Division of Clinical Immunology, Graduate School of Medical Sciences, Kitasato University, 1-15-1 Kitasato, Minami-ku, Sagamihara, Kanagawa 252-0373, Japan; 4 Division of Clinical Immunology, Graduate School of Medical Sciences, Kitasato University, 1-15-1 Kitasato, Minami-ku, Sagamihara, Kanagawa 252-0373, Japan; 5 Department of Laboratory Animal Science, Kitasato University School of Medicine, 1-15-1 Kitasato, Minami-ku, Sagamihara, Kanagawa 252-0373, Japan; 6 Division of Clinical Immunology, Graduate School of Medical Sciences, Kitasato University, 1-15-1 Kitasato, Minami-ku, Sagamihara, Kanagawa 252-0373, Japan; 7 R & D Center for Cell Design, Institute for Regenerative Medicine and Cell Design, Kitasato University School of Allied Health Sciences, 2) Division of Immunology, Kitasato University School of Allied Health Sciences, Kitasato 1-15-1, Sagamihara Minami-ku, Kanagawa 252-0373, Japan

**Keywords:** Parkinson’s disease, LRRK2, Transgenic mouse

## Abstract

**Background:**

*Leucine-rich repeat kinase 2 (LRRK2)* is the gene responsible for autosomal-dominant Parkinson’s disease (PD), PARK8, but the mechanism by which LRRK2 mutations cause neuronal dysfunction remains unknown. In the present study, we investigated for the first time a transgenic (TG) mouse strain expressing human LRRK2 with an I2020T mutation in the kinase domain, which had been detected in the patients of the original PARK8 family.

**Results:**

The TG mouse expressed I2020T LRRK2 in dopaminergic (DA) neurons of the substantia nigra, ventral tegmental area, and olfactory bulb. In both the beam test and rotarod test, the TG mice exhibited impaired locomotive ability in comparison with their non-transgenic (NTG) littermates. Although there was no obvious loss of DA neurons in either the substantia nigra or striatum, the TG brain showed several neurological abnormalities such as a reduced striatal dopamine content, fragmentation of the Golgi apparatus in DA neurons, and an increased degree of microtubule polymerization. Furthermore, the tyrosine hydroxylase-positive primary neurons derived from the TG mouse showed an increased frequency of apoptosis and had neurites with fewer branches and decreased outgrowth in comparison with those derived from the NTG controls.

**Conclusions:**

The I2020T LRRK2 TG mouse exhibited impaired locomotive ability accompanied by several dopaminergic neuron abnormalities. The TG mouse should provide valuable clues to the etiology of PD caused by the LRRK2 mutation.

## Background

*Leucine-rich repeat kinase 2 (LRRK2)* is the gene responsible for autosomal-dominant Parkinson’s disease (PD), PARK8, which originally has been defined by linkage analysis of a Japanese family (Sagamihara family) [1-4]. LRRK2 is a complex kinase consisting of LRR, ROC, COR, kinase, and WD40 domains [5]. The Sagamihara family patients have the I2020T mutation in the kinase domain [2]. Accumulated evidence suggests that LRRK2 may play a key role in axonal extension, autophagy, proliferation, and survival of neurons through its kinase activity [6-9]. In spite of the proposed mechanisms for neurodegeneration in vitro, the mechanism by which LRRK2 mutations affect DA neurons in patients and model animals in vivo is still far from conclusive.

As a mammalian PD model, transgenic (TG) mice expressing the R1441G mutation at the LRRK2 ROC domain reportedly show reduction of locomotive ability and diminished dopamine release [10]. The R1441C knock-in (KI) mouse, on the other hand, appears normal in steady-state, although a reduction of amphetamine-induced locomotor activity and impaired D2 receptor function have been observed [11]. Four different TG mouse lines expressing the G2019S mutation in the LRRK2 kinase domain, have been reported [12-15]. Two of them displayed increased ambulatory activity but the others did not. In terms of pathology, only one of them showed degeneration of DA neurons accompanied by abnormal autophagy, whereas two others showed increased tau-phosphorylation or promotion of tubulin polymerization associated with Golgi fragmentation. Temporal overexpression of G2019S LRRK2 in rat reportedly impairs dopamine reuptake, leading to enhanced locomotive activity [16].

In contrast to the extensive analysis of R1441G, R1441C, and G2019S TG mice, no LRRK2 TG rodent model with the I2020T mutation has ever been reported. G2019S and I2020T, despite being mutations affecting neighboring residues, have been known to have distinctive effects on the LRRK2 molecule, as reflected in kinase activity and susceptibility to post-translational degradation [17,18]. In *Drosophila*, TG flies expressing the I2020T LRRK2, or its homologue I1915T LRRK, have been reported to show either DA neuron loss leading to unusual locomotive activity or a decrease of neuromuscular junction boutons [19-21]. In the present study, we investigated a TG mouse strain expressing I2020T LRRK2 in DA neurons. The TG mouse exhibits impaired locomotive ability, a reduced striatum dopamine content, fragmented Golgi apparatus, and an elevated degree of tubulin polymerization. Furthermore, the tyrosine hydroxylase-positive (TH^+^) primary neurons of the TG mouse show increased vulnerability and shortened neurites.

## Results

### Generation of I2020T LRRK2 TG mice

**Figure 1:**
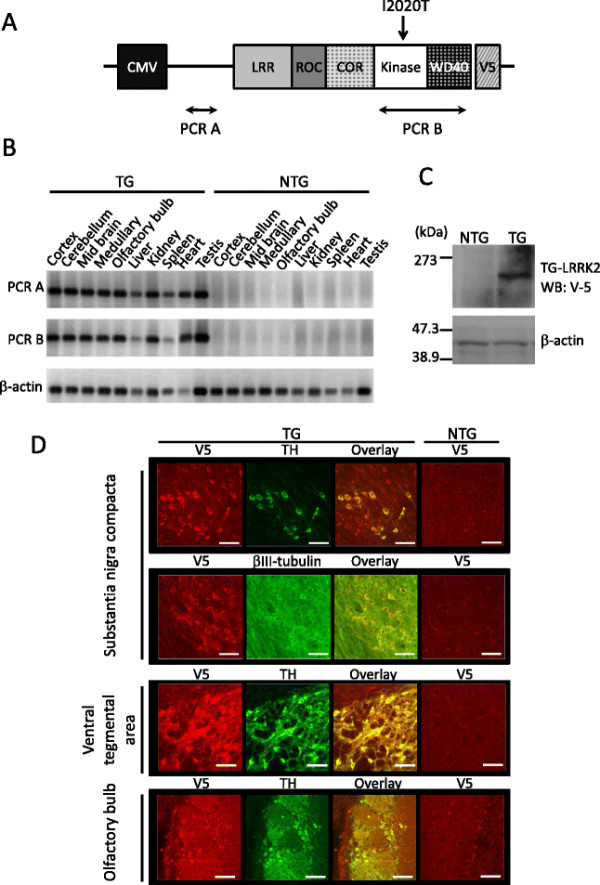
**Expression of I2020T LRRK2 in the TG mouse.** (**A**) Schematic representation of the LRRK2 cDNA transgene. (**B**) RT-PCR analysis of I2020T LRRK2 TG (line 41) and non-transgenic littermate mice. The amplified regions are shown in (A). (**C**) Western blotting analysis of whole brain. Lysates prepared from the whole brain of NTG and TG mice were subjected to Western analysis using anti V5-tag antibody. β-actin was used as a protein loading control. (**D**) Confocal immunostaining images of LRRK2 (V5), neurons (β-III tubulin), and dopamine neurons (TH) in the substantia nigra compacta, ventral tegmental area, and olfactory bulb of TG and NTG mice. Scale bar: 30 μm.

We obtained 9 independent mouse lines harboring the V5-tagged human LRRK2 cDNA with the I2020T mutation and single nucleotide polymorphisms (SNPs) of the Sagamihara family patients (Figure 1A). One of them (line 41) was found by genomic Southern analysis to harbor about 5 copies of the transgene at two insertion sites, one of which had multiple tandem insertions (Additional file 1. figures S1A and S1B). Although some other lines harbored more copy numbers than line 41, the latter line was the only line stably expressing the full-length I2020T LRRK2 in brain, and was therefore used throughout in this study. The line 41 TG mice appear healthy from birth and develop with normal weight and fertility, and live births show the expected Mendelian ratio. The TG mice expressed I2020T LRRK2 mRNA in all of the tissues analyzed (Figure 1B). Western analysis of whole brain using an anti-V5 antibody detected the I2020T LRRK2 full-length proteins (Figure 1C). Immunofluorescence staining of brain tissues using anti-V5, anti-TH, and anti-β-III tubulin antibodies indicated that the neuronal cells including TH^+^ neurons of the substantia nigra compacta (SNc), ventral tegmental area, and olfactory bulb expressed the I2020T LRRK2 proteins (Figure 1D). Quantitative PCR revealed that the level of expression of LRRK2 mRNA in the line 41 TG mice was about 1.5-, 1.4-, and 1.2-fold that of NTG control mice in the whole brain, striatum, and midbrain, respectively (Additional file 1. figure S2). Measurement of the immunofluorescence intensity of individual TH^+^-neurons in the substantia nigra with MJFF2 recognizing both human LRRK2 and mouse LRRK2 revealed that the level of LRRK2 protein expression in TH^+^-neurons of TG mice was about 1.3-fold that of TH^+^-neurons in NTG control mice (Additional file 1. figure S3).

### I2020T LRRK2 TG mice exhibit impaired locomotive ability

**Figure 2:**
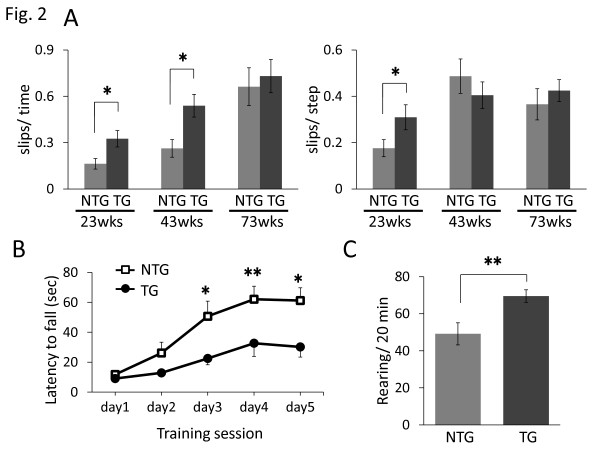
**Impaired locomotive ability of I2020T LRRK2 TG mice.** (**A**) Beam test. Each mouse was forced to walk on a narrow square beam (100 cm long, 5 mm wide). The time taken, the number of steps required to reach the platform, and the frequency of slips, were recorded. Left: number of slips normalized by time spent. Right: number of slips normalized by total steps: 23 weeks (NTG, n=13; TG, n=11), 43 weeks (NTG, n=13; TG, n=11), 73 weeks (NTG, n=6; TG, n=9). (B) Rotarod test. The time until the mouse fell from the rotating rod (16 rpm, 3 cm diameter) was recorded. The averages of three trials in one day are shown. The test was continued for 5 days (NTG, n=14; TG, n=11; 34 weeks). (C) Cylinder test. The frequency of rearing in the cylinder (9.5 cm diameter) during 20 min is shown (NTG, n=13; TG, n=11; 22 weeks). In all graphical representations, data are expressed as mean ± SEM and were assessed by Student’s *t* test at each time point; * *p*<0.05. ** *p*<0.01.

To assess the locomotive ability of I2020T LRRK2 TG mice, we subjected them to the beam test. While TG mice aged 23 weeks traversed the narrow beam, they exhibited slips more frequently (in terms of both per time and per step) than their non-TG (NTG) littermates (Figure 2A). This impaired locomotive ability was also observed in 43-week-old TG mice (slips per time), but was undetectable in 73-week-old TG mice. In the rotarod test, I2020T TG mice aged 34 weeks were unable to keep walking for as long as their NTG littermates during the training sessions on days 3, 4, and 5 (Figure 2B). Older TG mice (aged 42 and 59 weeks) showed a non-significant tendency to perform more poorly than NTG mice in the rotarod test (Additional file 1. figure S4). The TG mice exhibited normal wire-grasp ability and a normal skeletal muscle histology (data not shown), indicating that their impaired locomotive ability was not attributable to muscle deterioration. In the cylinder test, the I2020T TG mice exhibited a higher frequency of rearing than the NTG controls (Figure 2C; 22 weeks). The TG mice also showed a non-significant tendency to exhibit a higher frequency of rearing and grooming in the open-field test (Additional file 1. Figure S5). The I2020T TG mice did not show any significant difference in olfactory function from the NTG controls (Additional file 1. figure S6).

### Golgi apparatus fragmentation in DA neurons of I2020T LRRK2 TG mice

**Figure 3:**
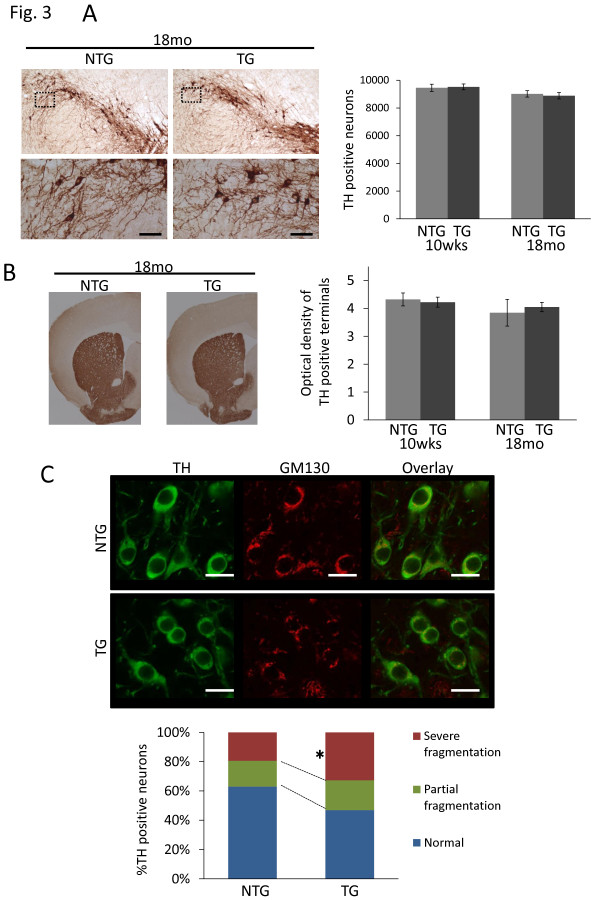
**TH-immunostaining and fragmentation of Golgi apparatus in I2020T LRRK2 TG mice.** (**A**) TH staining of DA neurons in the substantia nigra compacta (SNc). A series of midbrain sections from I2020T LRRK2 TG and NTG littermate mice of 18 months were stained with antibody against TH. Higher-magnification views of the boxed area show the morphology of TH-immunoreactive neurons. Scale bar: 50 μm. Right panel shows quantification of TH-immunoreactive neurons in the SNc by stereological counting. 10 weeks (NTG, n=3; TG, n=3), 18 months (NTG, n=3; TG, n=3). (**B**) TH staining of DA neurites in the dorsal striatum of TG and NTG littermate mice of 18 months. Right panel shows optical density analysis. 10 weeks (NTG, n=3; TG, n=3), 18 months (NTG, n=3; TG, n=3). (C) Immunostaining of TH and GM130 (cis-Golgi) in the SNc. TH-positive neurons were classified into three groups – normal, partial fragmentation, and severe fragmentation – depending on the degree of fragmentation of Golgi structures (50 neurons were counted for each mouse; NTG, n=3; TG, n=3; 10 weeks). Scale bar: 10 μm. Quantitative data are expressed as mean ± SEM and were analyzed by Student’s *t* test; *p<0.05.

Anatomic evaluation of the I2020T LRRK2 TG mouse brain with Nissl staining showed no obvious abnormalities in any area, including the cortex, midbrain, and cerebellum (data not shown). Immunohistochemical staining for TH revealed no loss of TH^+^ DA neurons in the SNc in either young (10 weeks) or old (18 months) TG mice, and the optical density of TH staining in the dorsal striatum showed no difference between NTG and TG mice (Figures 3A and 3B). Notably, immunofluorescence staining indicated that the I2020T LRRK2 TG mice had more TH^+^ DA neurons with severely fragmented Golgi apparatus than those of the NTG controls (Figure 3C; 10 weeks). Immunostaining using the anti-V5-tag antibody revealed that I2020T LRRK2 was located in the Golgi apparatus, suggesting that it may have a deleterious role in this location (Additional file 1. figure S7).

### Tubulin of the I2020T TG brain shows greater polymerization

**Figure 4:**
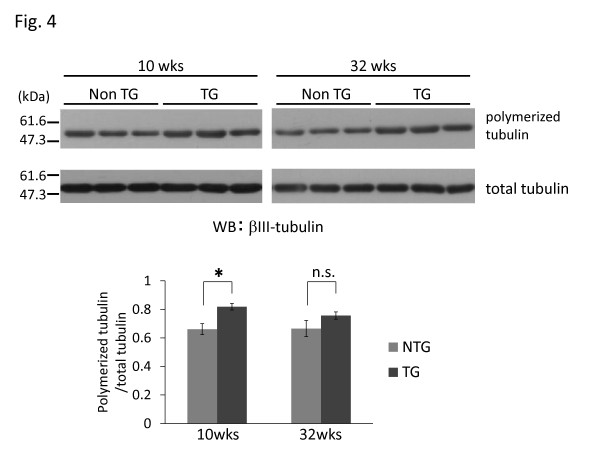
**Increased microtubule polymerization in I2020T LRRK2 TG mice.** Upper: Brain microtubules of I2020T LRRK2 TG and NTG littermate mice were subjected to in vitro polymerization assay and analyzed by Western blotting. 10 weeks (NTG, n=6; TG, n=6), 32 weeks (NTG, n=4; TG, n=4). Lower: The degree of tubulin polymerization expressed as mean ± SEM and were analyzed by Student’s *t* test at each time point; *p<0.05.

It has been shown that microtubules play a critical role in organization of the Golgi complex [22]. To examine whether microtubules of the I2020T LRRK2 TG brain have any unusual features, we performed an in vitro polymerization assay. Tubulin isolated from TG brains (10 and 32 weeks) exhibited an increased degree of polymerization – although not statistically significant at 32 weeks – in comparison with that isolated from the corresponding littermate NTG brains (Figure 4). To investigate whether tau phosphorylation affects microtubule polymerization status, Western analysis and immunostaining for phosphorylated Thr181, Ser202, Ser202/Thr205, Ser212/Thr214, Ser262, Ser396, and Ser404 were performed using TG mice aged 12 weeks. However, no particular difference was observed between the TG and NTG brains (data not shown). Immunostaining for phosphorylated α-synuclein (Ser129) and analysis of protein carbonyls as a general marker of oxidative damage in TG mice aged 22 weeks revealed no abnormal features in the TG brain (data not shown).

### Reduced striatal dopamine level in I2020T LRRK2 TG mice

**Figure 5:**
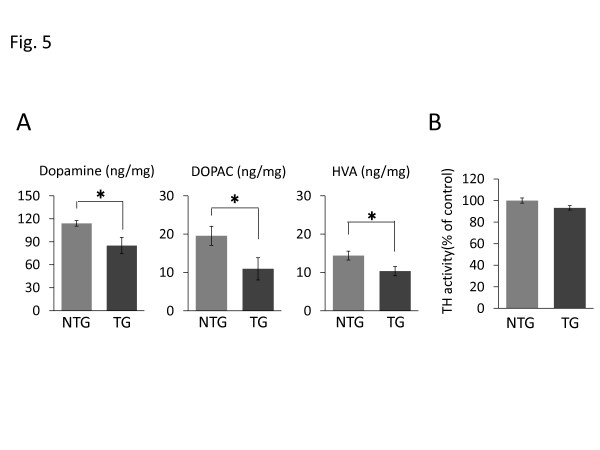
**Reduced striatal catecholamines in I2020T LRRK2 TG mice.** (**A**) Striatal concentrations of dopamine and its metabolites, DOPAC and HVA. Concentrations of catecholamines were determined by HPLC and electrochemical detection (NTG, n=6; TG, n=6; 10 weeks). (**B**) Striatal TH activity. The TH enzymatic activity was determined by measuring the amount of L-dopa. The vertical scales indicate the ratio of L-dopa in TG mice relative to that in NTG mice (NTG, n=3; TG, n=3; 10 weeks). Quantitative data are expressed as mean ± SEM and were assessed by Student’s *t* test; *p<0.05.

The concentrations of dopamine and its metabolites, 3,4-dihydroxyphenylacetic acid (DOPAC) and homovanillic acid (HVA), in the striatum of the I2020T LRRK2 TG mice were measured by high-performance liquid chromatography (HPLC). This revealed that the TG mice had significantly lower levels of striatal dopamine and its metabolites than the NTG controls (Figure 5A; 10 weeks). This low dopamine content, despite the lack of obvious DA neuron loss, may explain the impaired locomotive ability of the TG mice. The low dopamine content of the TG brain was not likely due to increased dopamine metabolism because the levels of DOPAC and HVA were also low, nor was it due to diminished TH enzymatic activity, as this was found to be equivalent to that in the NTG controls (Figure 5B; 10 weeks).

### Primary TH^+^ neurons of I2020T LRRK2 TG mice are vulnerable to apoptosis and have defective neurites

**Figure 6:**
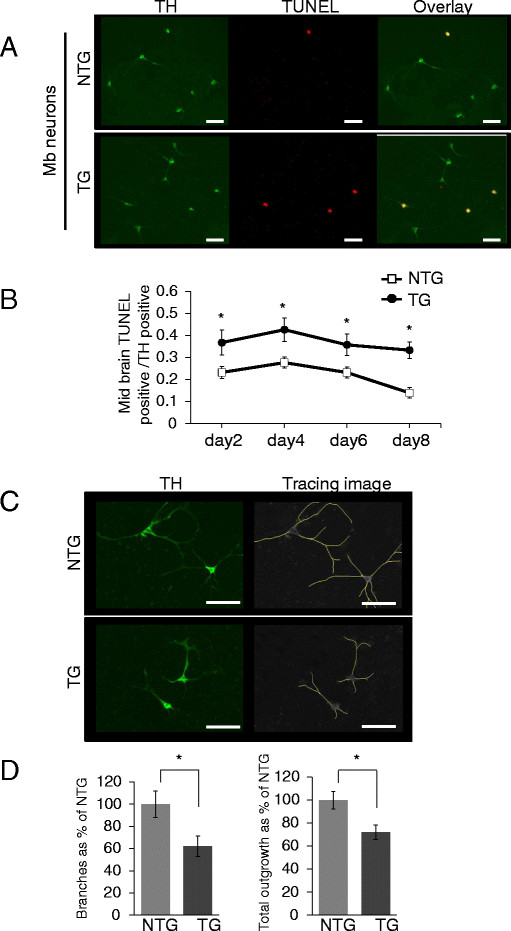
**Vulnerability and defective neurites of primary TH**^**+**^**neurons derived from I2020T LRRK2 TG brain.** (**A**) Anti-TH immunostaining of primary neurons prepared from the midbrain of I2020T LRRK2 TG and NTG mice. Apoptotic neurons were visualized by TUNEL assay. Scale bar: 30 μm. (**B**) Ratio of TUNEL-positive cells among TH^+^ neurons during culture. (**C**) Anti-TH immunostaining of primary neurons and the tracing image of neurites. Scale bar: 30 μm. (**D**) Left: number of neurite branches of TUNEL-negative TH^+^ neurons. Right: total neurite outgrowth of TUNEL-negative TH^+^ neurons (NTG, n=15; TG, n=15). Quantitative data are expressed as mean ± SEM and were assessed by Student’s *t* test at each time point; *p<0.01.

Finally, to understand the cellular characteristics of TH^+^ neurons of the I2020T LRRK2 TG mouse, we prepared primary neuron cultures from the fetal midbrain. TH^+^ neurons accounted for about 6 % of all primary midbrain neurons. During 8 days of culture, the primary TH^+^ neurons prepared from the TG brain exhibited a significantly higher ratio of TUNEL-positive apoptotic cells than those prepared from the NTG control (Figures 6A and 6B). Furthermore, morphological analysis of the TUNEL-negative TH^+^ neurons revealed that neurites of the TG-derived TH^+^ neurons had fewer branches and a decreased total outgrowth in comparison with those derived from the NTG controls (Figures 6C and 6D). These results suggested that although the adult TG brain has no obvious neuronal loss or morphological alteration, the TH^+^ neurons of the I2020T TG mouse have intrinsic vulnerability and neurite deficiency.

## Discussion

In the present study, we investigated for the first time a TG mouse strain expressing human LRRK2 with the I2020T mutation, which affects the kinase domain in the patients of the original PARK8 family (Sagamihara family). The I2020T TG mice exhibited several neurological abnormalities, such as impaired locomotive ability, DA neurons with a fragmented Golgi apparatus, a decreased striatal dopamine content, and an elevated degree of tubulin polymerization. In addition, the primary TH^+^ neurons derived from the TG brain showed high vulnerability and had neurites with defective characteristics. Our study provides the first in vivo evidence that expression of I2020T LRRK2 in mouse brain causes impaired locomotive ability and neurophysiological abnormality.

Importantly, the I2020T TG mice exhibited motor dysfunction in the beam test and rotarod test under physiological conditions, along with the decreased striatal dopamine content. On the other hand, it has been reported that animals showing striatal dopamine loss do not necessarily exhibit motor deficits in behavioral tests [23-25]. Although the beam test (narrow beam-traverse test) has been employed as a useful behavioral test for model animals with genetic and drug-induced PD [26-28], in the present study we further refined the experimental conditions of the test (width, form, and height of beam, etc.) to detect the abnormality present in the I2020T TG mouse. In addition, the improved rotarod test (high-position rotarod and continuous day trial) used in this study has been reported to have high sensitivity for detecting presymptomatic or early-stage PD, i.e., *Parkin*-KO mice showing no abnormality in the typical rotarod test reportedly exhibited an obvious motor deficit in the improved rotarod test [29], and model rats with presymptomatic PD induced by intracranial injection of 6-hydroxydopamine also exhibited a motor deficit in the improved rotarod test [30]. Thus, these sensitive behavioral tests appear to be capable of detecting the motor deficits in I2020T TG mice. On the other hand, neither the beam test nor the rotarod test detected a significant difference between aged TG and NTG mice. Further investigations including methodological refinements will be necessary before it can be proved that locomotive ability is restored with aging. In the cylinder test, the I2020T TG mice showed an increased frequency of rearing. This is in contrast to R1441G TG mice, which reportedly exhibit decreased rearing [10]. The TG mice also showed a non-significant tendency to exhibit a higher frequency of rearing and grooming in the open-field test.

We did not detect any obvious DA neuron loss in the I2020T TG mouse brain like that reported in most other LRRK2 TG mice, except for one G2019S TG line [15]. The I2020T TG brain did not form α-synuclein- or tau-positive aggregated materials. These neuropathological features may not be discordant from those of the Sagamihara patients harboring the I2020T mutation, whose postmortem brains have revealed only mild loss of DA neurons and no detectable Lewy bodies or neurofibrillary tangles [31,32]. On the other hand, the TG mouse expressing I2020T LRRK2 in the olfactory bulb had a sense of smell similar to that of control mice, whereas the Sagamihara patients show a degree of olfactory dysfunction ranging from slight impairment to anosmia [32].

The DA neurons of I2020T LRRK2 TG mice showed increased fragmentation of the Golgi apparatus. The structure of the Golgi apparatus is maintained by an appropriate microtubule polymerization status [22]. Microtubule-polymerizing reagents such as taxol and microtubule-depolymerizing reagent such as vinblastine reportedly disrupt the Golgi apparatus [33]. Our finding that brain tubulin in I2020T TG mice had a tendency to be excessively polymerized suggests that one of the mechanisms responsible for Golgi fragmentation could be impaired tubulin stability. These features of this TG mouse strain are very similar to those of one of the reported G2019S LRRK2 TG mouse strains showing a fragmented Golgi apparatus and an increased insoluble tubulin fraction [13]. Although we did not detect any alteration of tau phosphorylation in the LRRK2 TG brain, other microtubule-associated proteins such as CRMP-2, a known substrate for LRRK2 [34], could influence the polymerization of tubulin in the TG brain. Alternatively, the mutant LRRK2 might have a directly deleterious effect on the Golgi apparatus, because we found that I2020T LRRK2 is located there, in accord with other studies of mouse neurons and C. elegans (in this case, a LRRK2-homolog LRK-1) [35,36]. In any event, the Golgi fragmentation appears to have resulted in some sort of defective characteristic of DA neurons in I2020T TG mice, as has been reported in brain tissues of patients with various neurodegenerative diseases, those of animal PD models, and even in neurons at the pre-apoptotic stage [37-40].

In accordance with their impaired locomotive ability, I2020T TG mice exhibited a reduced striatal dopamine content. Analysis of the TH enzymatic activity and dopamine metabolites revealed that this low dopamine content might not be ascribable to either reduced synthesis or increased metabolism. It has been proposed that LRRK2 plays a key role in the trafficking of pre-synaptic vesicles by regulating membrane dynamics [41-43]. In the DA neurons of I2020T TG mice, fragmentation of the Golgi apparatus might hamper the maturation of some vesicle proteins, and hyper-polymerization of tubulin might disrupt their proper organization into membranes and vesicles [43,44]. Although the exact mechanism responsible for the reduced dopamine content of the I2020T TG striatum is unknown, a possible distortion of membrane/vesicle dynamics might affect the consumption and recycling of synaptic dopamine.

Although no significant neuronal loss was obvious in the adult brain up to 18 months old, the primary TH^+^ neurons derived from the I2020T TG midbrain showed a higher degree of apoptosis than those from the NTG controls, indicating that the I2020T mutation might confer intrinsic vulnerability on TH^+^ neurons. This finding is consistent with reports demonstrating that overexpression of I2020T LRRK2 in primary neurons induces neurotoxicity [46]. Also, we and other groups have demonstrated that neuroblastoma cell lines expressing I2020T LRRK2 are more susceptible to oxidative stress than those expressing wild-type LRRK2, although no consensus has been established regarding the molecular mechanism involved [19,46,47]. The neurites of primary TH^+^ neurons in the TG mouse also exhibited abnormal features, i.e., few branches and decreased outgrowth, consistent with one of the reported G2019S TG mouse strains whose cultured TH^+^ neurons also showed a reduction of neurite complexity [48]. These intrinsic defects detectable in primary TH^+^ neurons may play some role in the neural dysfunction observed in adult I2020T TG mice.

## Conclusions

We have established a TG mouse strain expressing the I2020T mutant human LRRK2. The TG mouse exhibits impaired locomotive ability and several neurological abnormalities. This strain should provide valuable clues to the etiology of PD caused by the LRRK2 mutation, as well as data relevant to the future development of therapeutic approaches.

## Methods

### Animals

Mice were housed in a light- and temperature-controlled room with water and food available ad libitum. For sacrifice, mice were euthanatized by cervical dislocation or exsanguination. All procedures had been approved by the Animal Experimentation and Ethics Committee of Kitasato University.

### Generation of I2020T LRRK2 transgenic mice

The V5-tagged human LRRK2 cDNA with the I2020T mutation and SNPs of the Sagamihara family patient has been described previously [8]. The 8,958-bp DNA fragment containing the CMV promoter and the LRRK2 cDNA including the tag was cut with Ahd I and Fsp I from the plasmid and microinjected into fertilized eggs of C57BL/6 J x C3H F1 female mice. The eggs were then transferred to the oviducts of pseudo-pregnant foster mothers of random-bred ICR. The founder TG mouse was back-crossed with the C57BL/6 J mouse more than 9 times, and their offspring were genotyped by polymerase chain reaction (PCR). The TG mice were backcrossed at least 9 times before being used for experiments. Methods for Southern blotting and RT-PCR are described in Additional file 2.

### Western Blotting

Mouse brains were homogenized in TNE buffer [10 mM Tris–HCl buffer (pH 7.6) containing 150 mM NaCl, 1 % Nonidet P-40, 1 mM EDTA, 1 mM phenylmethylsulfonyl fluoride, and protease inhibitor cocktail (Roche)] and kept gently agitated by slow rotation at 4 °C for 1 hour. The brain lysate was obtained by centrifugation at 13,000 rpm for 15 min at 4 °C, and its protein concentration was determined using BCA protein assay reagents (Thermoscientific). The lysates (40 μg) were subjected to sodium dodecyl sulfate polyacrylamide gel electrophoresis (SDS-PAGE) using 5-20 % gradient e-PAGEL (ATTO) or 10 % gels, and blotted onto polyvinylidene fluoride (PVDF) membranes. The membranes were blocked in 2 % skim milk or 2 % ECL Advance Blocking Agent (GE Healthcare) in phosphate-buffered saline (PBS)-0.1%Tween 20 for 60 min at room temperature and probed with the appropriate primary antibodies overnight at 4 °C. After incubation with secondary antibodies for 30 min at room temperature, protein bands were visualized using an ECL- or ECL-Advance Western Blotting Detection Kit (GE Healthcare).

### Immunostaining

Mice were subjected to flush-perfusion with heparinized saline, followed by perfusion-fixation with 4 % paraformaldehyde. The brains were removed, immersed in 4 % paraformaldehyde overnight, and subsequently in 30 % sucrose for 48 h at 4 °C. Brain sections 30 μm thick were subjected to H_2_O_2_-inactivation of endogenous peroxidase activity and treated with 2 % BSA in PBS-0.2 % Triton X-100 for 60 min at room temperature to block non-specific protein binding. For immunofluorescence staining, the tissue sections were incubated with rabbit polyclonal antibody against V5-tag (MBL), rabbit polyclonal antibody against TH (Millipore), mouse monoclonal antibody against β-III tubulin (R&D Systems), mouse monoclonal antibody against GM130 (BD Transduction Laboratories), and mouse monoclonal antibody against TH (Millipore) for 48 h at 4 °C, and subsequently with fluorescein isothiocyanate (FITC) or phycoerythrin (PE)-conjugated appropriate secondary antibodies for 120 min at room temperature. For TH-immunohistochemical analysis of the substantia nigra and striatum, a biotinylated secondary antibody against rabbit IgG was used together with an ABC kit (Vector Laboratories) for detection. Optical density was analyzed using NIH ImageJ software (http://rsbweb.nih.gov/ij/). Method for measurement of LRRK2 immuno fluorescence intensity in TH^+^-neurons is described in Additional file 2.

### In vitro polymerization of brain tubulin

Brain tissues were homogenized in low-salt reassembly buffer (RAB) [0.1 M Tris–HCl (pH 6.8), 0.5 mM MgSO_4_, 1 mM EGTA, and 2 mM dithiothreitol] at 4 °C, and centrifuged at 15,000 rpm for 1 h at 4 °C [49]. The supernatants were supplemented with 4 M glycerol and 1 mM GTP, and incubated for 90 min at 37 °C to polymerize the microtubules. After centrifugation at 15,000 rpm for 60 min at 37 °C, the resulting pellets were resuspended in 500μl RAB and incubated on ice for 30 min to depolymerize the microtubules. The polymerization/depolymerization cycle was repeated once, and the resulting microtubule material was resuspended in 250 μl RAB and subjected to Western analysis using the antibody against -III tubulin.

### Determination of catecholamines

The dorsal striatum was dissected out and quickly frozen in liquid nitrogen. Samples were homogenized in 500 μl of sample buffer for HPLC [0.2 M perchloric acid, 100 μM EDTA (pH 7.5)] and centrifuged at 15,000 rpm for 5 min at 4 °C. The supernatants were analyzed for dopamine, DOPAC, and HVA using HPLC coupled with electrochemical detection. Levels of DA, DOPAC, and HVA were determined by using standard curves, and normalized by tissue weight.

### TH activity assay

Striatal TH activity was determined as reported previously [50]. Briefly, the dorsal striatum was homogenized in 10 mM potassium phosphate buffer (pH 7.4), centrifuged at 3,000 rpm for 60 min at 4 °C, and the supernatant was mixed with an equal volume of reaction buffer [100 mM sodium acetic acid buffer (pH 6.0), 10 μg catalase, 1 mM NSD-1015, an inhibitor of aromatic L-amino acid decarboxylase, and 2 mM ferrous ammonium sulphate], and preincubated at 37 °C for 5 min. The reaction was started by adding 200 μM L-tyrosine and 10 mM (6*R*) tetrahydrobiopterin (BH_4_). After incubation for 10 min at 37 °C, the reaction was terminated by adding 100 μl of 0.1 M perchloric acid containing 0.4 mM sodium metabisulphite and 0.1 mM disodium EDTA. As the blank control, a similar reaction mixture containing D-tyrosine instead of L-isomer and 100 μM 3-iodo-L-tyrosine was used. The samples were subjected to HPLC and the amount of L-DOPA was measured.

### Primary neuron culture

Fetal mice at embryonic day 14.5-15.5 were obtained from the uterus and genotyped for the LRRK2 transgene. Fetal midbrain sections were dissected under a microscope and digested with papain at 37 °C for 20 min. The dispersed cells were suspended in growth medium [F-12 HAM (Sigma), B27 (Gibco), penicillin/streptomycin] and cultured on polyethyleneimine-coated cover slips (Sigma) placed in 24-well plastic tissue culture plates. Three days after plating, Ara C (Sigma) was added to inhibit the growth of glial cells and the medium was changed twice a week. TH^+^ neurons were identified by immunostaining. The numbers of branches and total outgrowth of neurites were analyzed using NIH ImageJ software (NeuronJ). Apoptotic cells were detected by TUNEL staining using an In Situ Cell Death Detection Kit (Roche).

### Behavioral tests

Beam test. First, mice were trained to walk on a wide beam (100 cm long, 25 mm wide) to motivate walking towards a dark platform. For the experimental test, each mouse was forced to walk along a narrow square beam (5 mm wide and 100 cm long, set at a height of 50 cm) to reach the platform. The time taken and the number of steps required to reach the platform, and the frequency of slips, were recorded.

*Rotarod test.* Each mouse was placed on a rubber-covered rod (3 cm in diameter) that was rotating at 16 rpm at a height of 50 cm (Shinano Seisakusho Co.). The length of time taken until the mouse fell from the rod was recorded (cut-off time: 180 s). The test was carried out three times per day and continued for 5 days.

*Cylinder test.* Each mouse was placed individually in an acrylic cylinder (25 cm high, 9.5 cm diameter) and video-recorded for 20 min. The frequency of vertical rearing by placing the forepaws on the wall was counted.

Methods for open-field test and olfactory testaredescribed in Additional file 2.

The numbers and genders of mice used for behavioral tests were summarized in Additional file 3. The other experiments were performed using only male mice.

*Statistical analysis.* All data are expressed as mean ± SEM. Significance of differences was assessed by Student’s *t* test.

## Competing interests

No competing interests for any relationships.

## Authors’ contributions

TM (the first author) designed and carried out the biochemical assay, immunostaining, and primary culture, and wrote the manuscript. SM and YS carried out the behavioral analysis and interpretation of the data. TM (the fourth author) carried out Western blotting analysis. SA prepared and bred the TG mice. EO prepared the I2020T-V5 LRRK2 construct. FO designed and interpreted the study overall, and drafted the manuscript. All authors read and approved the final manuscript.

## Supplementary Material

Additional file 1 **Figure S1. Genomic Southern analysis of the I2020T LRRK2 TG lines.** (A) Copy number analysis of 9 TG lines. TG mouse genomic DNA was cleaved with EcoRI and subjected to Southern analysis using a probe hybridizing with the middle portion of the LRRK2 insert. The intensity of the 3,404-bp fragment of LRRK2 cDNA introduced into the mouse genome was compared with that of a known amount of LRRK2 cDNA. NTG: non-transgenic negative control. (B) Chromosomal insertion-pattern analysis of TG line 41. Genomic DNA of TG line 41 was cleaved with Bgl II and EcoRI, and subjected to Southern analysis using a 3'-terminal region probe hybridizing with genomic DNA fragments having the insertion site-dependent size. The hybridization signals of 2,474 bp (Bgl II) and 2,310 bp (Eco RI) indicate tandem insertion, and the other signals indicate single-copy insertion. Genomic DNA of TG line 74 was used as a control giving a different insertion pattern, and that of C57BL/6 (B6) was employed as a negative control. **Figure S2. Analysis of I2020T LRRK2 mRNA expression.** RNA was isolated from the whole brain (Wb), striatum (St), and midbrain (Mb) region of TG line 41 and NTG control mice, and subjected to quantitative RT-PCR using primers annealing both human LRRK2 and mouse endogenous LRRK2. LRRK2 mRNA expression was normalized relative to that of GAPDH.**Figure S3. Measurement of LRRK2 immunofluorescence intensity in TH**^**+**^**-neurons.** The substantia nigra of TG and NTG control mice was subjected to double immunofluorescence staining with an anti-TH antibody and with MJFF2, recognizing both human LRRK2 and mouse LRRK2. The intensity of LRRK2 immunofluorescence in individual TH^+^-neurons (350 cells for TG and 533 cells for NTG) was measured using ImageJ software. **p<0.005. **Figure S4. Rotarod test for mice of different ages.** TG mice and their corresponding NTG littermates at different ages (34, 42, and 59 weeks) mice were subjected to the rotarod test for 5 continuous days. 34 weeks (NTG, n=14; TG, n=11), 42 weeks (NTG, n=11; TG, n=11), 59 weeks (NTG, n=14; TG, n=11). Data are expressed as mean ± SEM and were analyzed by Student’s *t* test at each time point. * *p*<0.05. ** *p*<0.01. **Figure S5. Open field tests.** Upper left: total distance walked. Upper right: percentage of time spent in the center. Lower left: number of rearing episodes. Lower center: number of grooming episodes. Lower right: number of stools produced (NTG, n=14; TG, n=11; 29 weeks). Student’s *t* test demonstrated no significant differences between TG and NTG mice for any of the measured parameters. **Figure S6. Olfactory test.** The time taken for mice to find hidden feed was recorded. As a control, feed was placed on top of the floor chips to make it visible, and the same trial was performed (NTG, n=6; TG, n=9; 14 weeks). Data are expressed as mean ± SEM. Student’s *t* test demonstrated no significant differences between TG and NTG mice. **Figure S7. Immunostaining of I2020T LRRK2 and the Golgi apparatus.** I2020T LRRK2 was stained with the anti-V5 tag antibody together with the anti-GM130 antibody (cis-Golgi). Arrows indicate the LRRK2 molecule co-localized with fragmented Golgi apparatus. Scale bar: 10 μm. Click here for file

Additional file 2 Materials and Methods. Click here for file

Additional file 3 **Table S3.** The numbers and genders of mice used for the behavioral tests. Click here for file
