# Supplementary material for: The I2020T Leucine-rich repeat kinase 2 transgenic mouse exhibits impaired locomotive ability accompanied by dopaminergic neuron abnormalities
Source: Mol Neurodegener. 2012 Apr 25;7:15. doi: 10.1186/1750-1326-7-15 (PMC3467184; doi:10.1186/1750-1326-7-15)
Supplement: Additional file 1 — Figure S1. Genomic Southern analysis of the I2020T LRRK2 TG lines. (A) Copy number analysis of 9 TG lines. TG mouse genomic DNA was cleaved with EcoRI and subjected to Southern analysis using a probe hybridizing with the middle portion of the LRRK2 insert. The intensity of the 3,404-bp fragment of LRRK2 cDNA introduced into the mouse genome was compared with that of a known amount of LRRK2 cDNA. NTG: non-transgenic negative control. (B) Chromosomal insertion-pattern analysis of TG line 41. Genomic DNA of TG line 41 was cleaved with Bgl II and EcoRI, and subjected to Southern analysis using a 3'-terminal region probe hybridizing with genomic DNA fragments having the insertion site-dependent size. The hybridization signals of 2,474 bp (Bgl II) and 2,310 bp (Eco RI) indicate tandem insertion, and the other signals indicate single-copy insertion. Genomic DNA of TG line 74 was used as a control giving a different insertion pattern, and that of C57BL/6 (B6) was employed as a negative control. Figure S2. Analysis of I2020T LRRK2 mRNA expression. RNA was isolated from the whole brain (Wb), striatum (St), and midbrain (Mb) region of TG line 41 and NTG control mice, and subjected to quantitative RT-PCR using primers annealing both human LRRK2 and mouse endogenous LRRK2. LRRK2 mRNA expression was normalized relative to that of GAPDH.Figure S3. Measurement of LRRK2 immunofluorescence intensity in TH+-neurons. The substantia nigra of TG and NTG control mice was subjected to double immunofluorescence staining with an anti-TH antibody and with MJFF2, recognizing both human LRRK2 and mouse LRRK2. The intensity of LRRK2 immunofluorescence in individual TH+-neurons (350 cells for TG and 533 cells for NTG) was measured using ImageJ software. **p<0.005. Figure S4. Rotarod test for mice of different ages. TG mice and their corresponding NTG littermates at different ages (34, 42, and 59 weeks) mice were subjected to the rotarod test for 5 continuous days. 34 weeks (NTG, n=14; TG, n=11), [file 1750-1326-7-15-S1.docx]

**Additional file. Materials and Methods**

*Southern blotting*

To estimate the copy number of the transgene, TG mouse genomic DNA was cleaved with EcoRI, and electrophoresed on 0.7% agarose gel. After alkali-vacuum transfer to a nylon membrane, the DNA was hybridized with alkaline phosphatase-labeled LRRK2 probes (1,110 bp) that had been prepared by amplification of the ROC domain of LRRK2 cDNA. The signal was detected with CDP-Star Detection Reagent (GE Healthcare) and compared with that of a known amount of LRRK2 cDNA. To obtain information on the chromosomal insertion sites, the genomic DNA was cleaved with Bgl II and EcoRI, and hybridized with a 3'-terminal region probe (627 bp) that would hybridize with DNA fragments having the insertion site-dependent size.

*Reverse transcription-polymerase chain reaction (RT-PCR)*

Mouse tissues were homogenized in TRIzol Reagent (Invitrogen) and RNA was isolated in accordance with the manufacturer’s [instructions](javascript:goWordLink(%22instructions%22)). The concentration of RNA was determined from the optical density. Synthesis of cDNA was performed using the ThermoScript RT-PCR System (Invitrogen). For identification of the transgene, two regions of human LRRK2-cDNA were amplified with the following primers: forward A 5'-AGCGTTGACGATAAGCATTG-3', reverse A 5'-GGGATTGAAGTTTTGATGACC-3', forward B 5'-CGAACATCTGTTGAGAAGGGC-3', and reverse B 5'-CCGGTACGCGTAGAATCGAGACCG-3'. β-actin was amplified as a control using following primers: forward 5'-GGTGACGAGGCCCAGAGCAAGAGA-3' and reverse 5'-CGACCAGAGGCATACAGGGACAGC-3'. Quantitative polymerase chain reaction was performed using SYBR Green PCR Master Mix and a 7500 Real Time PCR System (Applied Biosystems). LRRK2 primers designed to anneal both mouse and human LRRK2 were as follows: forward 5'-CAGCAGGACAAAGCCAGCCTC-3' and reverse 5'-GCAATGATGGCAGCATTGGGATAC-3'. The threshold cycle (Ct) value was normalized with the glyceraldehyde-3-phosphate dehydrogenase (GAPDH) gene.

*Measurement of LRRK2 immunofluorescence intensity in TH^+^-neurons*

The substantia nigra of TG and NTG control mice was subjected to double immunofluorescence staining with a mouse monoclonal antibody against TH (Millipore) and a rabbit monoclonal antibody, MJFF2 (Epitomics), recognizing both human LRRK2 and mouse LRRK2, and subsequently with fluorescein FITC or PE-conjugated appropriate secondary antibodies. The fluorescence intensity of PE for LRRK2 staining in individual TH^+^-neurons was measured using ImageJ software.

*Open-field test*

Each mouse was placed individually in a 40 cm x 40 cm white box for 20 min with a surface illumination intensity of 212 lux. The movement of the mouse was video-recorded and analyzed using ImageJ software (O’Hara & Co., Ltd.). The total distance, the percentage of time spent in the center, the number of rearing episodes, the number of grooming episodes, and the number of stools produced were analyzed.

*Olfactory test*

Mice were fasted for 24 hours before the test. Individual mice were transferred to a new cage in which feed was hidden under the chips covering the floor. The time taken for the mice to find the hidden feed was recorded. As a control, feed was placed on top of the floor chips to make it visible, and the same trial was performed.
