# Supplementary material for: The I2020T Leucine-rich repeat kinase 2 transgenic mouse exhibits impaired locomotive ability accompanied by dopaminergic neuron abnormalities
Source: Mol Neurodegener. 2012 Apr 25;7:15. doi: 10.1186/1750-1326-7-15 (PMC3467184; doi:10.1186/1750-1326-7-15)
Supplement: Additional file 2 — Materials and Methods. [file 1750-1326-7-15-S2.docx]

**Additional file. Figure legend**

**Figure S1. Genomic Southern analysis of the I2020T LRRK2 TG lines.**

(A) Copy number analysis of 9 TG lines. TG mouse genomic DNA was cleaved with EcoRI and subjected to Southern analysis using a probe hybridizing with the middle portion of the LRRK2 insert. The intensity of the 3,404-bp fragment of LRRK2 cDNA introduced into the mouse genome was compared with that of a known amount of LRRK2 cDNA. NTG: non-transgenic negative control. (B) Chromosomal insertion-pattern analysis of TG line 41. Genomic DNA of TG line 41 was cleaved with Bgl II and EcoRI, and subjected to Southern analysis using a 3'-terminal region probe hybridizing with genomic DNA fragments having the insertion site-dependent size. The hybridization signals of 2,474 bp (Bgl II) and 2,310 bp (Eco RI) indicate tandem insertion, and the other signals indicate single-copy insertion. Genomic DNA of TG line 74 was used as a control giving a different insertion pattern, and that of C57BL/6 (B6) was employed as a negative control.

**Figure S2. Analysis of I2020T LRRK2 mRNA expression.**

RNA was isolated from the whole brain (Wb), striatum (St), and midbrain (Mb) region of TG line 41 and NTG control mice, and subjected to quantitative RT-PCR using primers annealing both human LRRK2 and mouse endogenous LRRK2. LRRK2 mRNA expression was normalized relative to that of GAPDH.

**Figure S3. Measurement of LRRK2 immunofluorescence intensity in TH^+^-neurons.**

The substantia nigra of TG and NTG control mice was subjected to double immunofluorescence staining with an anti-TH antibody and with MJFF2, recognizing both human LRRK2 and mouse LRRK2. The intensity of LRRK2 immunofluorescence in individual TH^+^-neurons (350 cells for TG and 533 cells for NTG) was measured using ImageJ software. **p<0.005.

**Figure S4. Rotarod test for mice of different ages.**

TG mice and their corresponding NTG littermates at different ages (34, 42, and 59 weeks) mice were subjected to the rotarod test for 5 continuous days. 34 weeks (NTG, n=14; TG, n=11), 42 weeks (NTG, n=11; TG, n=11), 59 weeks (NTG, n=14; TG, n=11). Data are expressed as mean ± SEM and were analyzed by Student’s *t* test at each time point. * *p*<0.05. ** *p*<0.01.

**Figure S5. Open field tests.**

Upper left: total distance walked. Upper right: percentage of time spent in the center. Lower left: number of rearing episodes. Lower center: number of grooming episodes. Lower right: number of stools produced (NTG, n=14; TG, n=11; 29 weeks). Student’s *t* test demonstrated no significant differences between TG and NTG mice for any of the measured parameters.

**Figure S6. Olfactory test.**

The time taken for mice to find hidden feed was recorded. As a control, feed was placed on top of the floor chips to make it visible, and the same trial was performed (NTG, n=6; TG, n=9; 14 weeks). Data are expressed as mean ± SEM. Student’s *t* test demonstrated no significant differences between TG and NTG mice.

**Figure S7. Immunostaining of I2020T LRRK2 and the Golgi apparatus.**

I2020T LRRK2 was stained with the anti-V5 tag antibody together with the anti-GM130 antibody (cis-Golgi). Arrows indicate the LRRK2 molecule co-localized with fragmented Golgi apparatus. Scale bar: 10 μm.
