# Supplementary material for: The I2020T Leucine-rich repeat kinase 2 transgenic mouse exhibits impaired locomotive ability accompanied by dopaminergic neuron abnormalities
Source: Mol Neurodegener. 2012 Apr 25;7:15. doi: 10.1186/1750-1326-7-15 (PMC3467184; doi:10.1186/1750-1326-7-15)
Supplement: Additional file 3 — Table S3. The numbers and genders of mice used for the behavioral tests. [file 1750-1326-7-15-S3.pptx]

## Slide 1
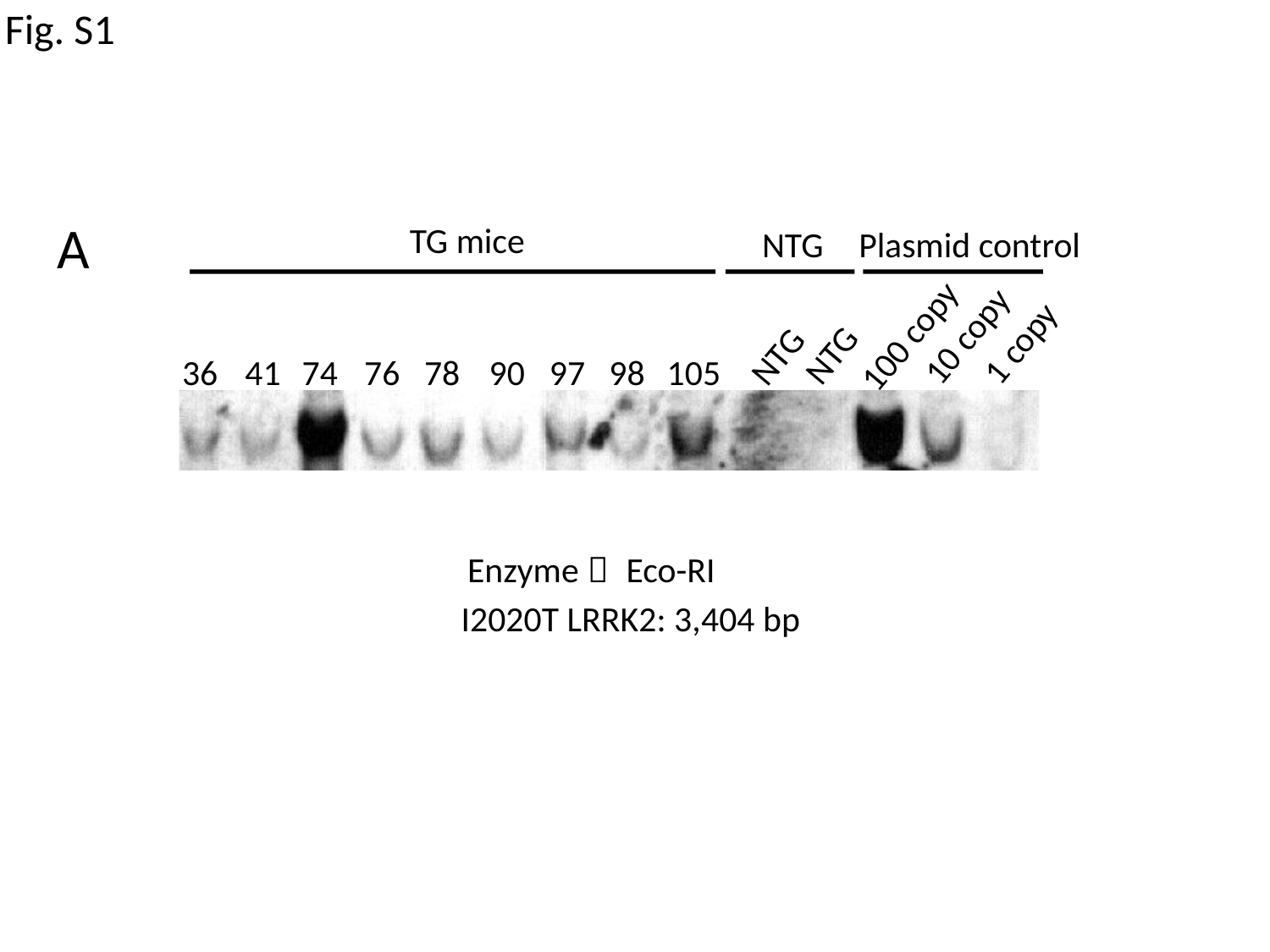

Fig. S1
A
 TG mice
 NTG
 Plasmid control
10 copy
100 copy
1 copy
NTG
 NTG
36
41
74
 76
 78
 90
 97
 98
 105
Enzyme： Eco-RI
I2020T LRRK2: 3,404 bp

## Slide 2
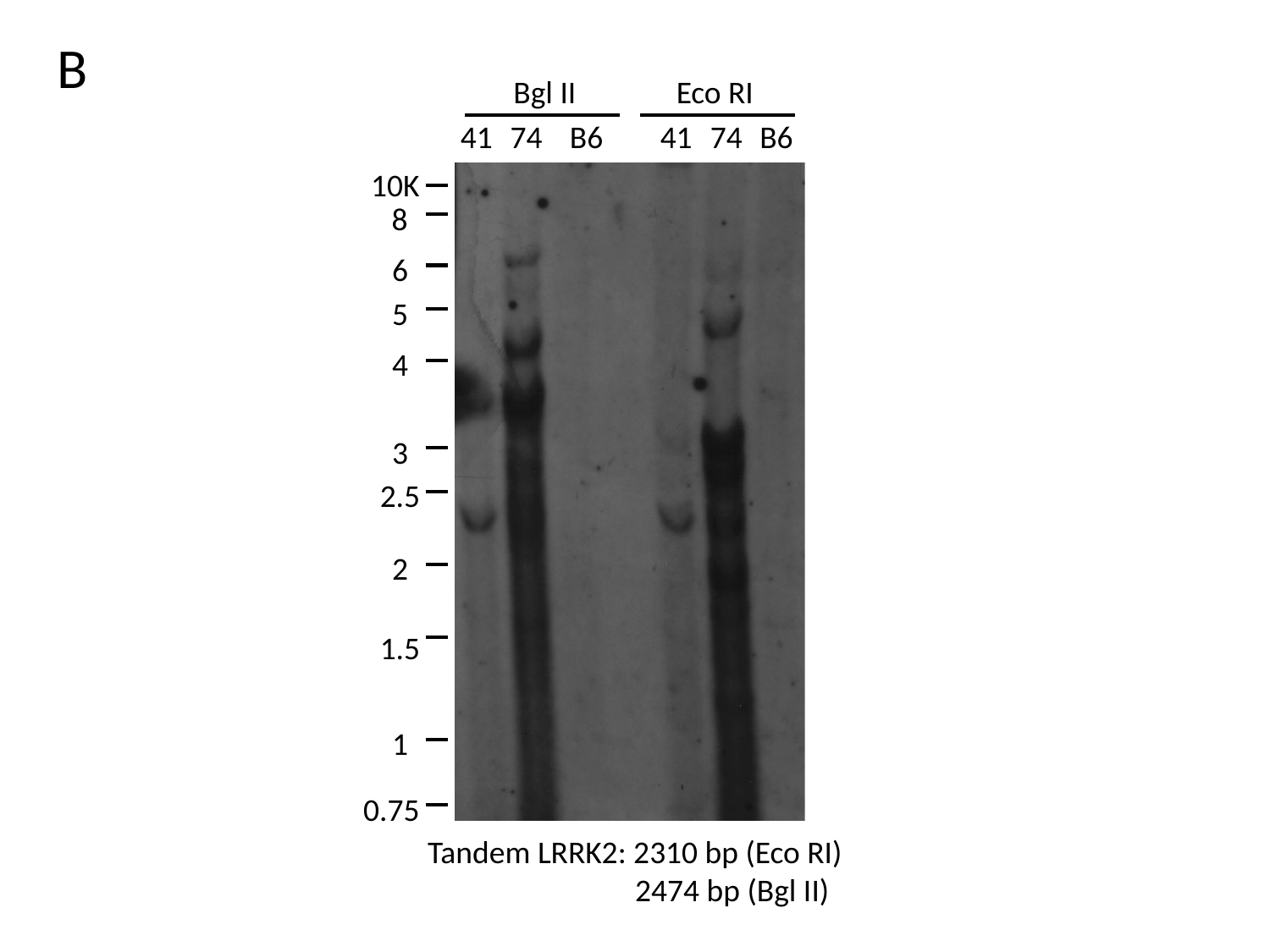

B
Bgl II
Eco RI
41
74
B6
41
74
B6
10K
8
6
5
4
3
2.5
2
1.5
1
0.75
Tandem LRRK2: 2310 bp (Eco RI)
 2474 bp (Bgl II)

## Slide 3
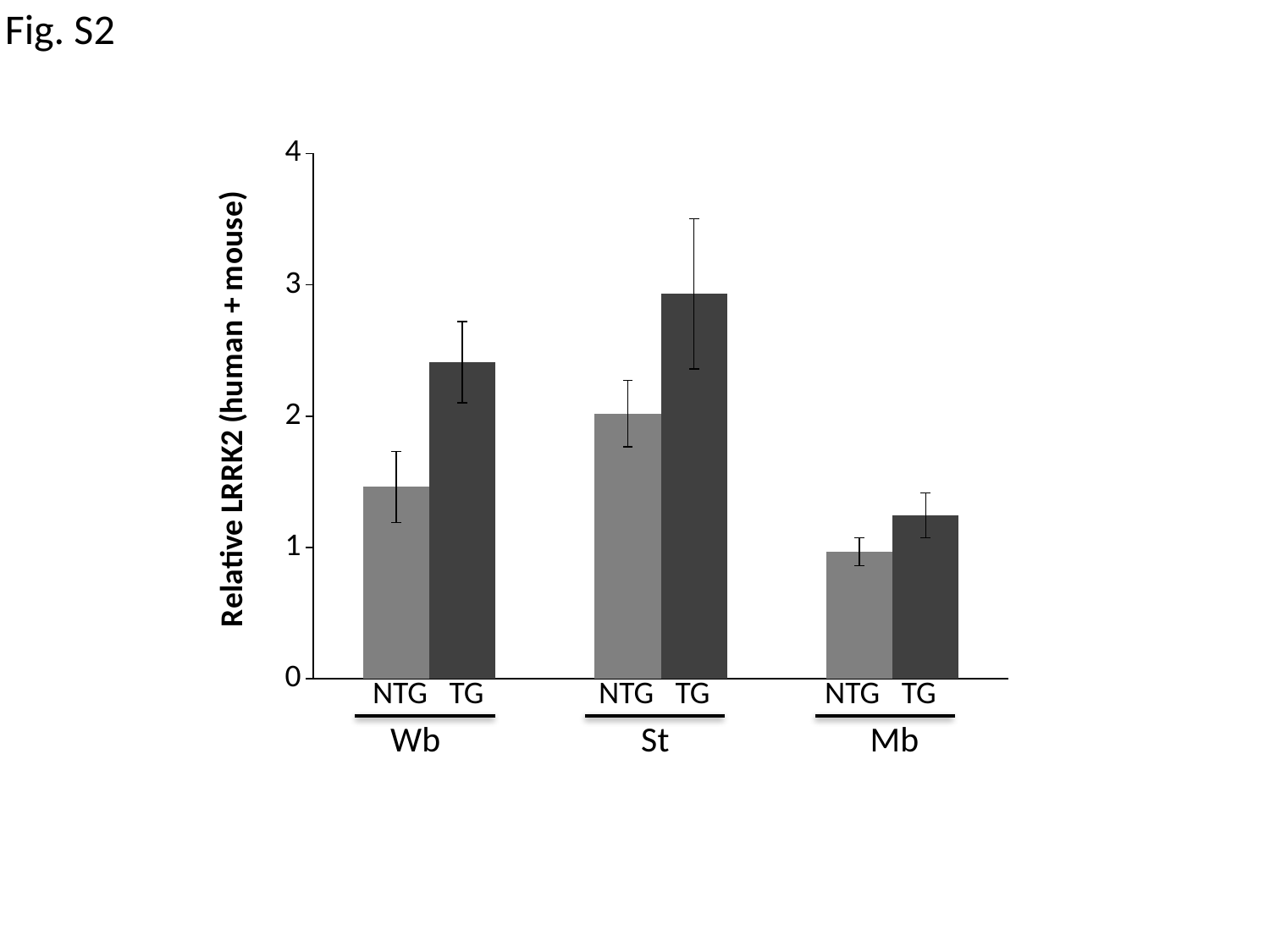

Fig. S2
### Chart
| Category | NTG | TG |
|---|---|---|
| Wb | 1.46024555438434 | 2.411006811004811 |
| St | 2.0182968854622207 | 2.931343908038718 |
| Mb | 0.966803226969924 | 1.2455937567556234 |NTG TG NTG TG NTG TG
Wb St Mb

## Slide 4
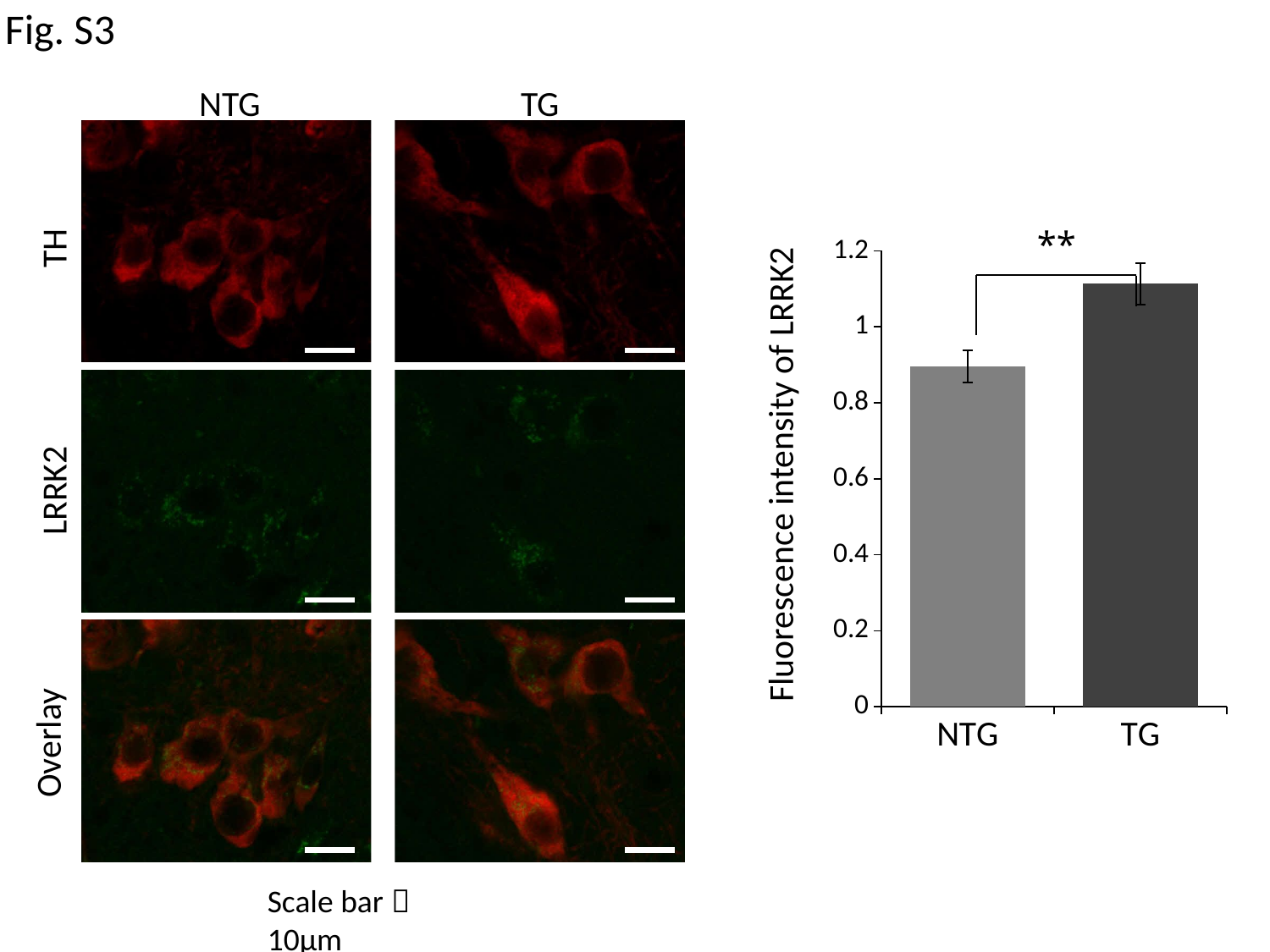

Fig. S3
NTG
TG
**
TH
### Chart
| Category | |
|---|---|
| NTG | 0.8960919324577893 |
| TG | 1.1129161904761902 |
Fluorescence intensity of LRRK2
LRRK2
Overlay
Scale bar：10μm

## Slide 5
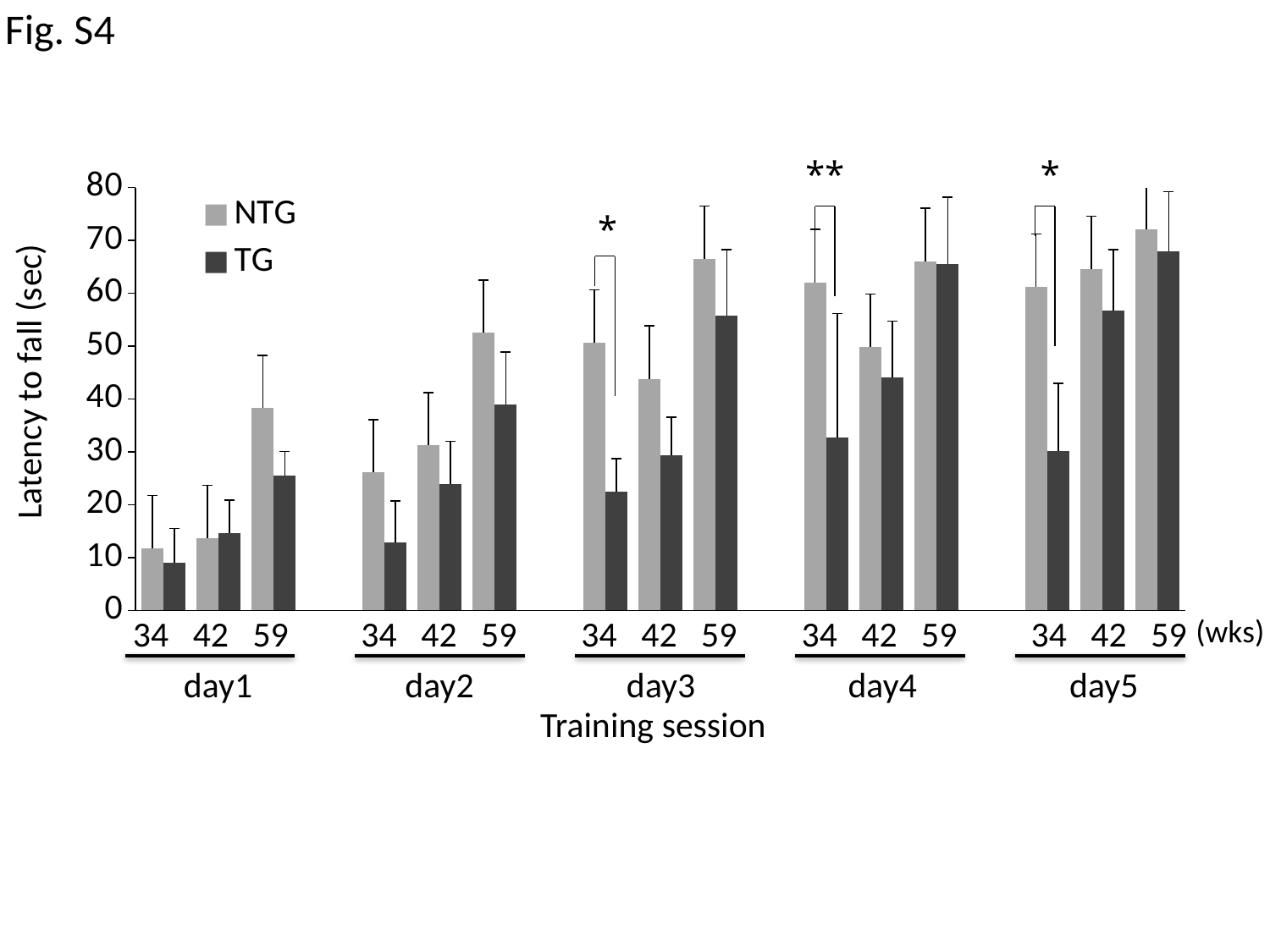

Fig. S4
**
*
### Chart
| Category | | |
|---|---|---|*
(wks)
34 42 59 34 42 59 34 42 59 34 42 59 34 42 59
 day1 day2 day3 day4 day5
Training session

## Slide 6
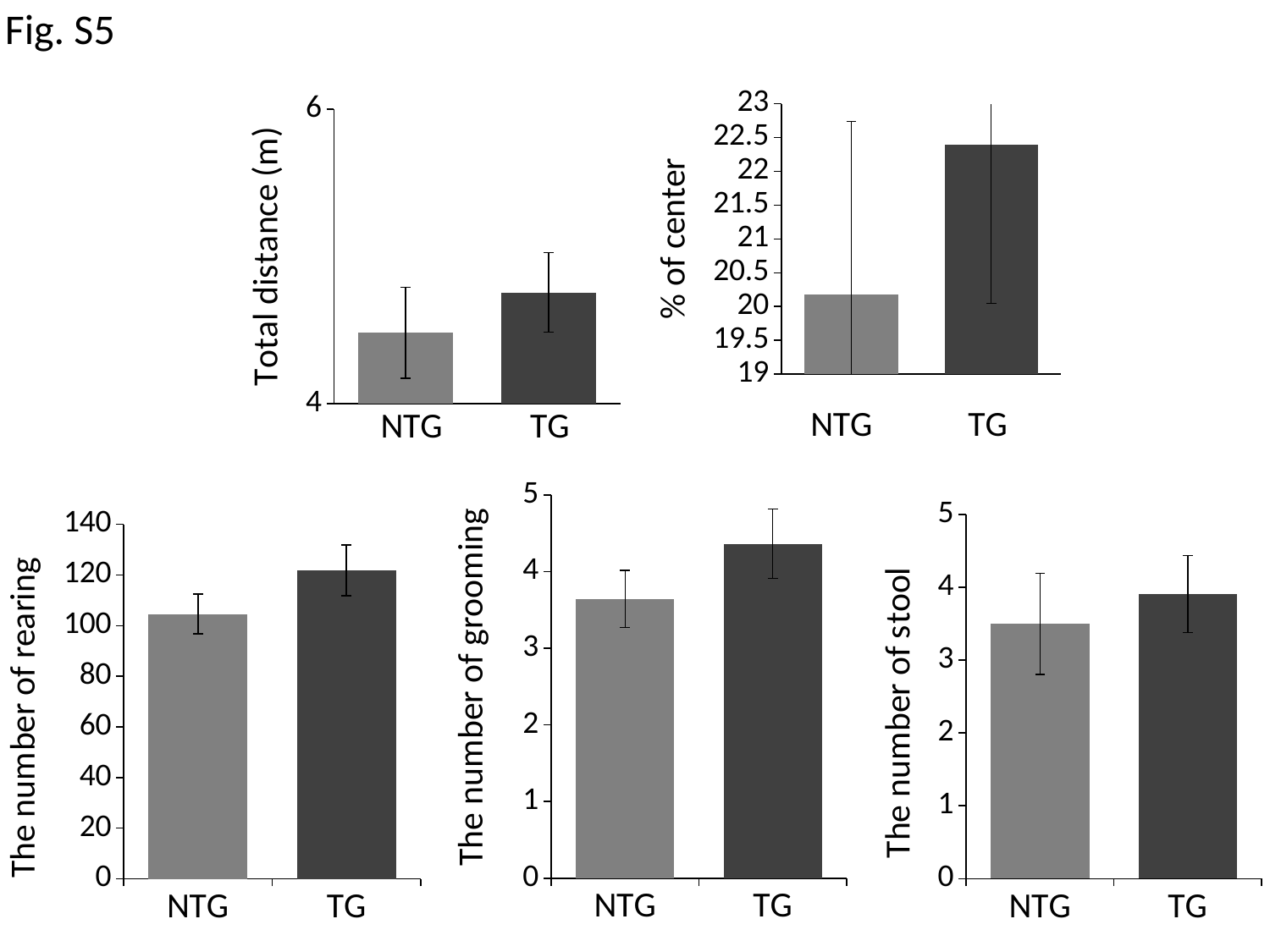

Fig. S5
### Chart
| Category | |
|---|---|
### Chart
| Category | |
|---|---|
### Chart
| Category | |
|---|---|
| NTG | 3.6428571428571432 |
| TG | 4.363636363636361 |
### Chart
| Category | |
|---|---|
| NTG | 3.5 |
| TG | 3.9090909090909087 |
### Chart
| Category | |
|---|---|
| NTG | 104.5714285714283 |
| TG | 121.8181818181812 |

## Slide 7
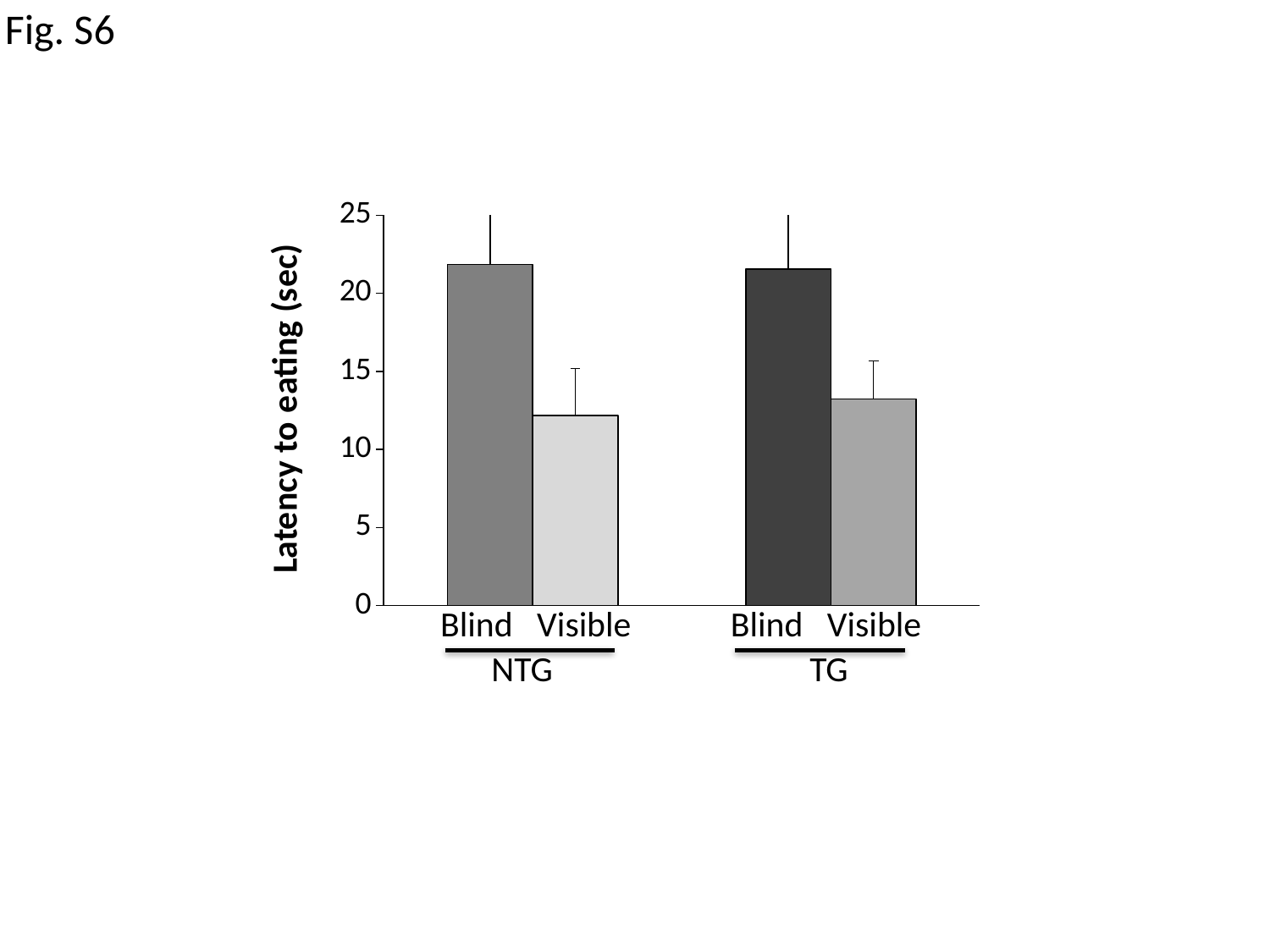

Fig. S6
### Chart
| Category | buried | surface |
|---|---|---|
| nonTG | 21.83333333333319 | 12.166666666666696 |
| TG | 21.55555555555556 | 13.22222222222222 |Blind Visible
Blind Visible
NTG TG

## Slide 8
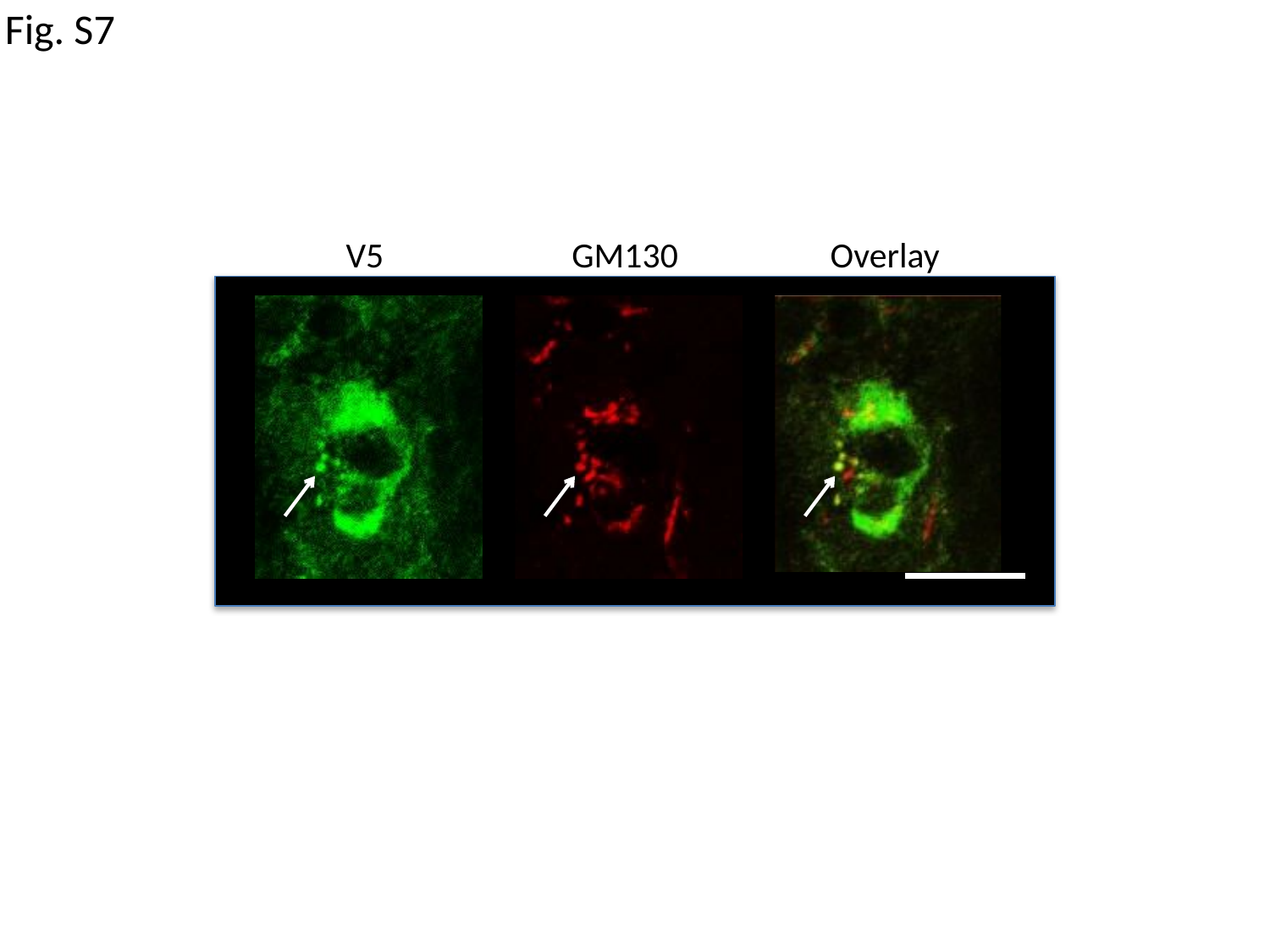

Fig. S7
V5
GM130
Overlay
